# Identification of gene expression changes associated with the initiation of diapause in the brain of the cotton bollworm, *Helicoverpa armigera*

**DOI:** 10.1186/1471-2164-12-224

**Published:** 2011-05-11

**Authors:** Bin Bao, Wei-Hua Xu

**Affiliations:** 1 State Key Laboratory of Biocontrol, School of Life Sciences, Sun Yat-Sen (Zhongshan) University, Guangzhou 510275, China

## Abstract

**Background:**

Diapause, a state of arrested development accompanied by a marked decrease of metabolic rate, helps insects to overcome unfavorable seasons. *Helicoverpa armigera *(Har) undergoes pupal diapause, but the molecular mechanism of diapause initiation is unclear. Using suppression subtractive hybridization (SSH), we investigated differentially expressed genes in diapause- and nondiapause-destined pupal brains at diapause initiation.

**Results:**

We constructed two SSH libraries (forward, F and reverse, R) to isolate genes that are up-regulated or down-regulated at diapause initiation. We obtained 194 unique sequences in the F library and 115 unique sequences in the R library. Further, genes expression at the mRNA and protein level in diapause- and nondiapause-destined pupal brains were confirmed by RT-PCR, Northern blot or Western blot analysis. Finally, we classified the genes and predicted their possible roles at diapause initiation.

**Conclusion:**

Differentially expressed genes at pupal diapause initiation are possibly involved in the regulation of metabolism, energy, stress resistance, signaling pathways, cell cycle, transcription and translation.

## Background

Environmental changes are an obvious source of stress for an organism. Insects inhabiting variable environments employ a number of adaptations to survive adverse conditions. Developmental arrest, called diapause in insects, is one evolutionary adaptation utilized to endure unfavorable conditions [1]. As a strategy for surviving unfavorable environmental conditions, diapause can occur in various developmental stages including egg, larva, pupa or adult, resulting in a programmed arrest of development coupled with other physiological changes [2]. Diapause is a dynamic process consisting of several successive phases: pre-diapause, diapause, and post-diapause, and each phase may comprise some sub-phases, e.g., the diapause phase is divided into diapause initiation, maintenance, and termination [3]. The hormonal regulation of diapause has been well defined, but the molecular mechanism of diapause is unclear. Using pulse labeling combined with 2-dimensional electrophoresis and elimination hybridization, changes in protein synthesis [4] and gene expression [5] were firstly identified in the diapausing pupal brain of *Sarcophaga crassipalpis*, suggesting that diapause is a unique developmental pathway rather than a simple shutdown of gene expression [6]. Suppression subtractive hybridization (SSH) has been used to evaluate diapause-specific gene expression in *Culex pipiens *[7] and *S. crassipalpis *[8]. Recently, a systemic investigation of transcript profiling of nondiapause and diapause pupae has been conducted using microarray technique in *S. crassipalpis *[9]. In addition, proteomic method has been used to identify differentially expressed proteins in the brains of *S. crassipalpis *[10] and *Helicoverpa armigera *[11]. Many genes and proteins related to diapause have been identified, but differentially expressed genes during diapause initiation are rarely reported [6]. It is yet unknown why individual insects can switch from direct development to arrested development.

The cotton bollworm *H. armigera *(Har), an agriculturally important pest, enters pupal diapause for survival in winter. After pupation, the diapause-destined pupae will enter diapause within 7--8 days, because day 9 of pupae can not develop towards adults, even though these pupae are incubated in a suit conditions. The physiological characteristics of diapause are observed on day 3 pupae, such as low ecdysone titer and unmoved eyespots, so differentially expressed genes as diapause instructions may be issued at an earlier pupal stage. Thus, we focused on gene expression in day 1 and 2 of pupae. As the programmable center of diapause, the brain is the most important organ to release instructions for diapause initiation [6]. To understand the molecular mechanism of diapause initiation, we searched for differentially expressed genes during pupal diapause initiation in *H. armigera *by using SSH. Meanwhile, the differentially expressed genes in nondiapause individuals were also investigated to search those genes expressed at low level in diapause-destined individuals.

## Results

### General statistics from two SSH libraries

Two subtracted cDNA libraries enriched in diapause- or development-correlative genes were constructed by using SSH. One was the F (forward) library expected to be enriched in diapause up-regulated cDNAs. The F library was obtained using the mRNAs from the diapause-destined pupal brains of days 1--2 after pupation as the "tester", and mRNAs from the nondiapause pupal brains of days 1--2 as the "driver". The other is the R (reverse) library to enrich cDNAs up-regulated in nondiapause-destined (or down-regulated in diapause-destined) pupal brain by reversing the "tester" and "driver" mRNAs.

**Figure 1:**
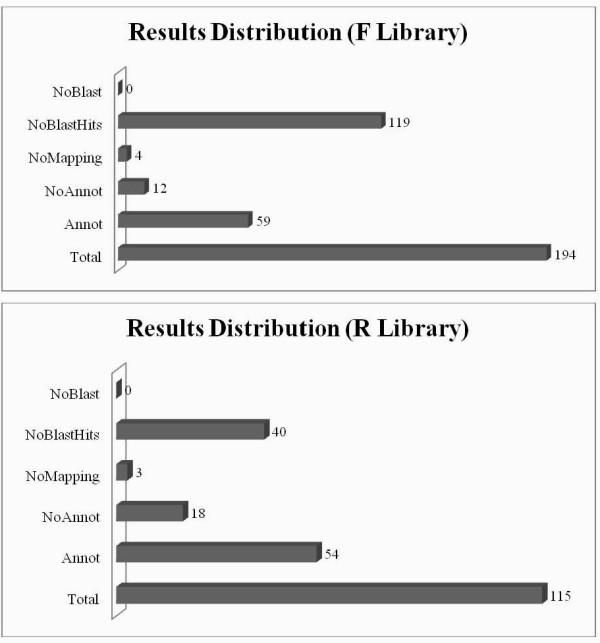
**Gene distribution from two SSH libraries**. In the F library, diapause-type pupae were used as the "tester", and nondiapause pupae as the "driver" to detect highly expressed genes in diapause-type pupae. By contrast, the "tester" and "driver" were reversed in the R library to detect highly expressed genes in nondiapause-type pupae. The Arabic numerals represent the number of unique sequence at each step of the annotation process. NoBlast, no blast results; NoAnnot, no annotation; Annot, annotation. Data analysis and visualization of results were performed by the Blast2GO software.

A total of over 1000 clones were randomly picked and subjected to colony PCR, and sequencing was carried out for 220 and 130 cDNA clones selected at random from the F and R libraries, respectively. After removing poor quality sequences, we finally obtained 194 unique sequences in the F library and 115 unique sequences in the R library. All the sequences (309) were compared against available databases to find similarities with known sequences. We carried out dynamic translation (blastx), and only matched sequences with an E-value lower than 10^-03 ^were considered to be homologous sequences. Sequences with an E-value higher than 10^-03 ^were labeled undescribed. Homologous sequences accounted for 38.7% of the sequences in the F library and 65.2% in the R library. The homologous sequences are shown in Additional File 1. Among the homologous sequences, 12 in the F library (12/75, 16.0%) and 18 in the R library (18/75, 24.0%) lacked annotation. Additionally, the number of no mapping sequences was four in the F library and three in the R library, respectively. All of these data are summarized in Figure 1.

### Gene ontology analysis

Most genes isolated from the two libraries have not been identified, so that we here define these genes as putatively up-regulated genes from the F library and putatively down-regulated genes from the R library as shown in Table 1.

**Table 1:** Classification of transcripts of known genes

Seq. Name	Seq. Description	Length(bp)	eValue	Similarity	Anotation
**A. Metabolism & Energy**					

*HarDP-A209*	enolase	325	9.73E-55	99.20%	Glycolysis
*HarDP-B843*	atp synthase f0 subunit 6	183	7.47E-15	94.70%	ATP synthesis
*HarDP-B170*	cytochrome c oxidase subunit ii	656	1.94E-65	93.80%	respiratory chain
*HarDP-B1356*	mitochondrial cytochrome c oxidase subunit 7c	647	1.19E-19	68.10%	generation of precusor metabolic and energy
**HarNP-1172**	fructose-1,6 -bisphosphatase	1130	5.18E-157	88.10%	gluconeogenesis
**HarNP-475**	aconitase	475	1.62E-14	87.45%	TCA
**HarNP-491**	malate synthase	449	1.57E-41	66.15%	Glyoxylate cycle
**HarNP-1261**	apolipoprotein d	1219	1.84E-88	61.35%	lipid metabolic process
**HarNP-1246**	lipase	1204	4.22E-61	57.80%	Lipid degradation

**B. Stress Resistence**					

***HarDP-A355***	heat shock protein 70	304	4.96E-51	98.70%	defense response
***HarDP-C556***	ferritin	514	1.09E-51	82.00%	antioxidation
***HarDP-C941***	ferritin light chain	899	7.25E-74	72.15%	antioxidation
***HarDP-B1408***	mn superoxide dismutase	624	2.60E-69	83.75%	antioxidation
***HarDP-A22***	glutathione s-transferase	234	1.43E-10	57.05%	antioxidation
***HarDP-A112***	bombyrin	330	1.98E-20	65.15%	antioxidation
***HarDP-A345***	rad23 homolog b (cerevisiae)	400	9.49E-34	69.80%	DNA repair
***HarDP-C509***	integrator complex subunit 3	467	3.28E-47	66.20%	DNA repair

**C. Signal**					

*HarDP-C672*	rac serine threonine kinase (Akt1)	630	1.70E-07	53.00%	AKT1, insulin signaling
**HarNP-1301**	ca2+ calmodulin-dependent protein kinase II	1260	1.66E-76	96.60%	longterm memory
**HarNP-138**	arginine kinase	138	2.41E-10	99.70%	promote growth

**D. Cell Cycle**					

**HarNP-668**	cyclin-dependent kinase 8	626	1.20E-90	92.15%	cell cycle
**HarNP-387**	80 kda mcm3-associated protein	345	5.45E-34	72.15%	DNA replication licensing factor
**HarNP-284**	gtp-binding nuclear protein ran	242	1.32E-40	99.00%	modulates both spindle and nuclear envelope assembly
**HarNP-831**	MCM9	789	4.85E-65	65.75%	DNA replication
**HarNP-1044**	Septin 2	1002	3.43E-30	47.45%	Required for the progression through mitosis
**HarNP-798**	transcription factor dp-2	756	1.61E-46	74.05%	involved in cell cycle regulation or DNA replication

**F. Transcription & Translation**					

*HarDP-C1098*	cg8378 cg8378-pa	1056	4.59E-20	45.90%	transcription repressor
*HarDP-A589*	ubiquitin-like protein smt3(SUMO)	517	5.91E-45	97.20%	transcription regulation
**HarNP-730**	pleiomorphic adenoma gene 1	688	6.44E-13	48.00%	transcription activitor
**HarNP-642**	elongation factor 1 delta	642	3.49E-64	71.65%	translation
**HarNP-418**	oocyte zinc finger protein xlcof22	376	1.88E-10	55.05%	transcription regulation
**HarNP-905**	Reptin	863	1.44E-06	87.50%	transcirption regulation

Transcripts were classified according to the function of homologous genes in other species. The putatively up-regulated genes at diapause initiation are shown in italics, and the putatively down-regulated genes are shown in bold

**Figure 2:**
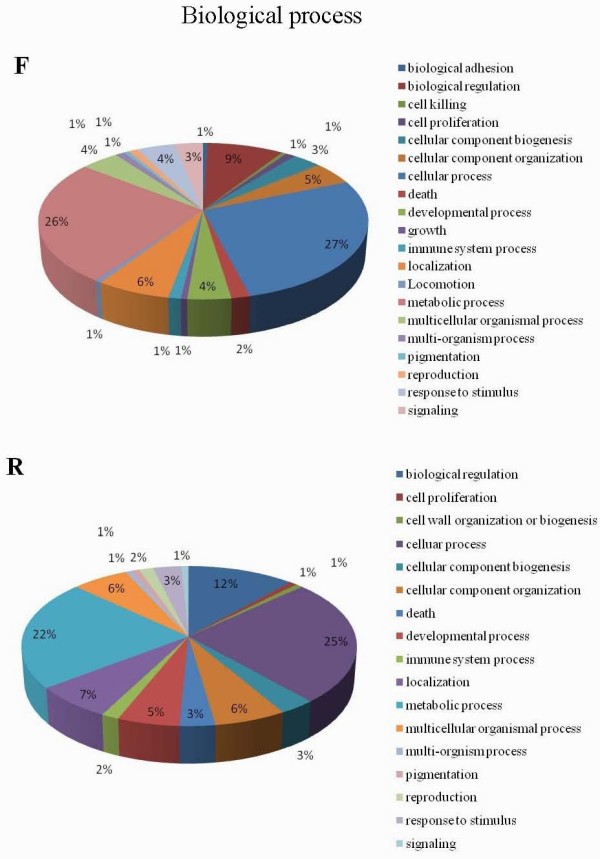
**Classification of the unique sequence from the SSH according to Gene Ontology criteria**. Gene Ontology analysis was carried out on the transcripts isolated from the two SSH libraries. The biological process combined graph was made based on ontology level 2.

Gene ontology analysis was carried out using the blast2go program [12]. Sequences were classified into the three ontology categories: biological process (Figure 2 and Additional File 2), molecular function and cellular component (Additional File 3). In the category "biological process", the most frequent process was cellular process (27% in the F library and 25% in the R library), followed by metabolic process (26% in the F library and 22% in the R library) and biological regulation (9% in the F library and 12% in the R library). In the category "molecular function", binding was the most frequent activity (36% in the F library and 47% in the R library), followed by catalytic activity (31% in the F library and 34% in the R library). Significant differences were observed in structural molecular activity (20% in the F library and 6% in the R library) and transcription regulator activity categories (1% in the F library and 6% in the R library). In the last category, "cellular component" the most frequent activity was macromolecular complex (25% in the F library and 18% in the R library), and membrane-enclosed lumen (3% in the F library and 8% in the R library) appeared to be different between the two SSH libraries.

### Identification of differentially expressed transcripts

**Figure 3:**
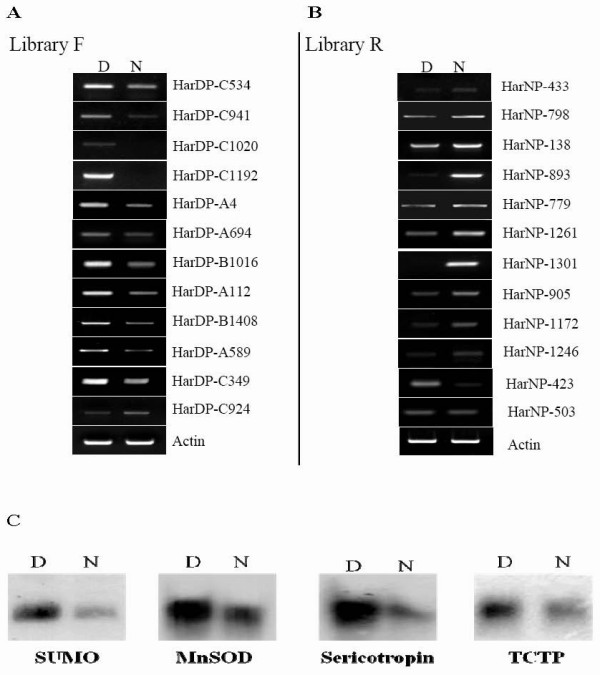
**Identification of differentially expressed transcripts**. Total RNA was extracted from day 1--2 brains of diapause- and nondiapause-destined pupae. Expression of 12 transcripts selected from the F (A) and R libraries (B) was analyzed by RT-PCR, with actin as a control. Four transcripts from the F library (C) were measured by Northern blot analysis. N, nondiapause-destined individuals; D, diapause-destined individuals. The results are representative of two independent experiments.

To test the reliability of the two SSH libraries, we randomly selected 12 genes from each library to investigate their expression levels in diapause- and nondiapause-destined brains at the early pupal stage by RT-PCR. In the F library, 11 transcripts were expressed highly in diapause-destined individuals, with HarDP-C924 being the exception (Figure 3A). In the R library, 10 transcripts were expressed highly in nondiapause pupae, with HarNP-423 and HarNP-503 being the exceptions (Figure 3B). Furthermore, the levels of four transcripts from the F library were confirmed by Northern blot analysis. As shown in Figure 3C, their expression was higher in diapause-destined pupae. These results show that the two SSH libraries are reliable.

### Expression patterns at diapause initiation

**Figure 4:**
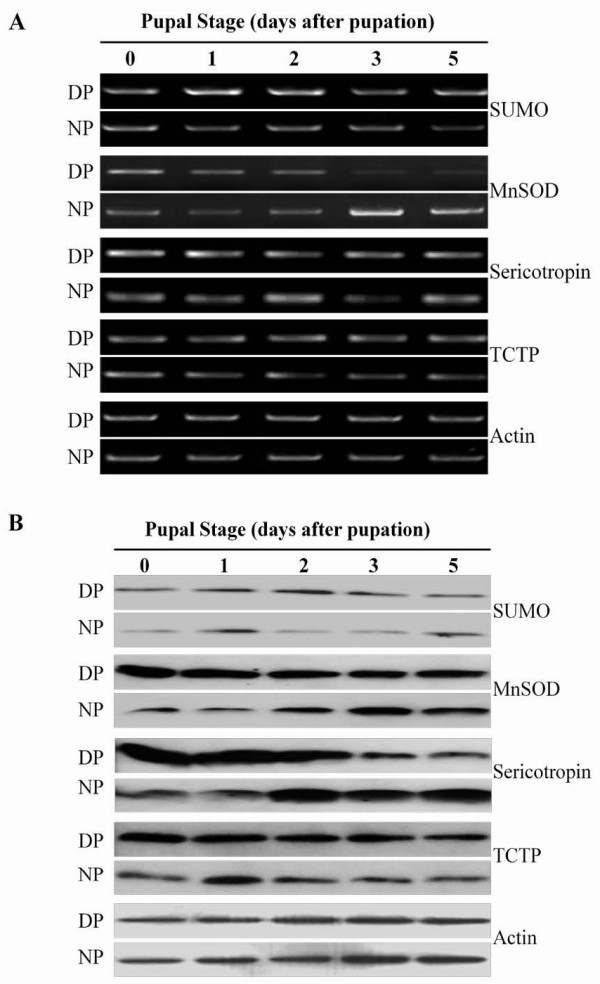
**Developmental expression patterns of genes isolated by SSH**. Expression pattern of four genes during the early stage of *Helicoverpa armigera *pupae. Total RNA and protein were extracted from brains and SGs or brain--SG complexes of diapause- and nondiapause-destined pupae. (A) Expression of genes measured by RT-PCR. The cDNAs of the four genes were amplified by PCR with 20--24 cycles; actin was amplified with 20 cycles. (B) Western blot analysis. Total protein (20 μg) was separated and incubated with polyclonal antibodies (Har-SUMO, 1:1000; Har-MnSOD, 1:3000; Har-Sericotropin, 1:2000; Har-TCTP, 1:3000). The numbers (0--5) represent the days after pupation, and actin was used as an internal standard. NP, nondiapause-destined pupae; DP, diapause-destined pupae.

To obtain some clues about the functions of the genes from the SSH library, the expression patterns of four selected genes in the brain were investigated during early pupal development by RT-PCR and Western blot analysis. The four genes encoded ubiquitin-like protein smt3 (SUMO, HarDP-A589), Mn superoxide dismutase (MnSOD, HarDP-B1408), sericotropin (HarDP-B1016) and translated controlled tumor protein (TCTP, HarDP-A694), which were assessed by Northern blot analysis above (Figure 3C). All four mRNAs were expressed higher during early pupal development in diapause-destined individuals, especially SUMO from day 1 to day 2, MnSOD from day 0 to day 2, sericotropin from day 0 to day 1, and TCTP from day 1 to day 5 (Figure 4A). The four protein levels reflected their mRNA levels (Figure 4B). Apparently, high expression of these genes at the early pupal stage is likely to be associated with pupal diapause initiation.

## Metabolism and energy

Nine genes, including four high- and five low-expression genes, were assigned to the metabolism and energy category (Table 1A). Two enzymes, aldolase (HarDP-C349) and enolase (HarDP-A209), which were up-regulated during diapause initiation, participate in glycolysis. In contrast, an enzyme fructose-1,6-bisphosphatase (FBP) (HarNP-1172), which is important in gluconeogenesis, was down-regulated at diapause initiation.

Aconitase (HarNP-475) and malate synthase (HarNP-491), which are important components of the tricarboxylic acid (TCA) cycle, are down-regulated at diapause initiation. Additionally, a set of transcripts (HarDP-B843, HarDP-B170 and HarDP-B1356) encoding proteins involved in ATP generation were up-regulated at diapause initiation. ATP synthase f0 subunit 6 (HarDP-B843) is a key component of ATP synthase. Cytochrome *c *oxidase subunit 2 (HarDP-B170) and cytochrome *c *oxidase subunit 7C (COX7C, HarDP-B1356) are two components of the respiratory chain in mitochondria.

Two genes related to lipid metabolism were found in the R library: HarNP-1261 and HarNP-1246 were down-regulated at diapause initiation. Apolipoprotein D (HarNP-1261) is closely associated with the enzyme lecithin:cholesterol acyltransferase and is involved in lipoprotein metabolism. Lipase (HarNP-1246) participates in the lipid degradation process.

## Stress resistance

Eight genes were assigned to the stress resistance category (Table 1B), all up-regulated at diapause initiation. Hsp70 (HarDP-A355) acts as a molecular chaperone to protect cellular proteins from denaturation and contributes to the cold tolerance of insects [13]. Another group of transcripts that was up-regulated at diapause initiation was related to antioxidation: ferritin (HarDP-C556), ferritin light chain (HarDP-C941), MnSOD (HarDP-B1408), glutathione S-transferase (GST, HarDP-A22) and bombyrin (HarDP-A112). The last two transcripts activated in response to stress are related to DNA repair: Rad23 (HarDP-A345) and integrator complex subunit 3 (HarDP-C509). Rad23 functions in UV-damaged DNA repair post-replication, and integrator complex subunit 3 is a component of the sensor of ssDNA (SOSS) complex, which is required for efficient homologous recombination-dependent repair of double-strand breaks [14].

## Signaling pathway

Several signaling pathways are involved in the regulation of developmental arrest, such as the guanylyl cyclase pathway, TGFβ-like pathway, insulin-like pathway, and steroid hormone pathway [15]. In this study, the transcription of the Akt gene (HarDP-C672) was up-regulated. Akt is an important protein in the insulin-like pathway (Table 1C). In contrast, calmodulin protein kinase II (CaMK II) (HarNP-1301) and arginine kinase (ArgK, HarNP-138) are down-regulated during diapause initiation. Calmodulin-dependent signaling is required for development, and CaMK II is a key member of this signaling pathway. ArgK is a phosphotransferase that catalyzes the reaction between L-arginine and ATP to produce L-phospho-arginine and ADP, and it functions in the regulation of ATP level, as creatine kinase in vertebrates [16].

## Cell cycle

Six transcripts down-regulated at diapause initiation were cell cycle regulators (Table 1D). Cyclin-dependent kinase (CDK) 8 (HarNP-668) is a member of the CDK family, which are important regulators of cell cycle progression. CDK8 is also a coactivator involved in regulated gene transcription of nearly all RNA polymerase II--dependent genes. The 80-kDa mcm3-associated protein (Har-NP387) interacts with MCM3, which is a factor that allows the DNA to undergo a single round of replication per cell cycle and is required for DNA replication and cell proliferation [17]. GTP-binding nuclear protein ran (Har-NP284) is involved in chromatin condensation and cell cycle control. MCM9 (Har-NP831), as a DNA replication licensing factor, participates in cell cycle regulation. Septin 2 (Har-NP1044) is required for the progression through mitosis [18]. Transcription factor dp-2 (TFDP2, HarNP-798) can stimulate E2F-dependent transcription and promote the transcription of a number of genes whose products are involved in cell cycle regulation or in DNA replication [19].

## Transcription and translation

Six genes related to transcription and translation were also found in the two SSH libraries (Table 1E). Two genes, CG8378 (HarDP-C1098), which is predicted to have transcriptional repressor activity, and SUMO (HarDP-A589), which always represses the activity of transcription factors, were up-regulated at diapause initiation. In contrast, four genes were down-regulated in diapause-type pupae: Pleomorphic adenoma gene 1 (HarNP-730) is a transcription factor whose activation results in up-regulation of target genes, such as Insulin-like growth factor (IGF) [20]. Elongation factor 1 delta (HarNP-642) facilitates the events of translational elongation, resulting in promotion of protein biosynthesis. Oocyte zinc finger protein xlcof22 (HarNP-418) functions in transcriptional regulation. Reptin (HarNP-905) acts as a transcriptional activator, and also as an essential cofactor for the normal function of Myc, so it is required for cellular proliferation and growth [21].

## Discussion

The insect brain is the center of developmental control and serves as the repository of the diapause program [22]. In pupal diapause species, photoperiodic signal is perceived by larval brain during diapause induction. Then gene expression changes affected by photoperiod are first present in diapause preparation phase which follows diapause induction to regulate specific metabolism for diapause [3,6,22]. It is well known that after pupation, a shut-down of prothoracicotropic hormone (PTTH) in the brain and ecdysteroids in the prothoracic gland cause diapause initiation [6]. Meola and Adkisson demonstrated that the shut-down of PTTH is found in day 0 of pupal brain of *Helicoverpa zea*, a closely related species to *H. armigera *[23]. Thus, these differentially expressed genes isolated from the two libraries in day 1-2 pupal brain of *H. armigera *for diapause initiation are in response to hormones, but not photoperiodic signal.

In *H. armigera*, the photosensitive stage for diapsuse induction is from 5^th ^instar to early stage of 6^th ^instar. This is little different compared to *H. armigera *population from Okayama (Japan), whose photosensitive stage for diapause induction is the early fifth instar [24]. After pupation, *H. armigera *diapause-type pupae are transferred into L14:10D photoperiod, all pupae will enter diapause, and all pupae will develop without diapause even if nondiapause-type pupae are transferred into L10:14D photoperiod (data not shown). Apparently, photoperiod regime does not affect pupal diapause or development.

The most remarkable characteristic of insect diapause is strong metabolic suppression. For example, in diapausing pupae of the flesh fly, *Sarcophaga argyrostoma*, the metabolic rate is approximately 90% lower than in nondiapause counterparts [25]. Therefore, diapause was thought to represent a shutdown in gene expression. However, Joplin et al. [4] and Flannagan et al. [5] demonstrated that diapause should be a unique developmental pathway rather than a simple shutdown of gene expression. Recently, the proteomic analysis of the brain at diapause initiation has been reported, suggesting that the expression of many diapause-specific genes in the brain accompanies certain down-regulated genes [10,11]. Thus, identification of diapause-associated genes at diapause initiation is the first step to understand the complex process of diapause. In the present paper, we isolated 304 diapause-specific mRNAs from *H. armigera *brain using SSH, and the subset of these genes with sequences similar to known genes in GenBank were classified according to their functions. Furthermore, we evaluated their mRNA expression at diapause initiation by RT-PCR and Northern blot analysis, and investigated the expression patterns of four important genes by RT-PCR and Western blot analysis, showing that these genes may be associated with diapause initiation.

From the SSH F library, we found a high percentage of undescribed sequences (61.3%). Some sequences may correspond to 3' or 5' untranslated regions (UTRs), so it is impossible to find their homologues in protein databases. However, most of these undescribed sequences can be classified as novel genes related to *H. armigera *pupal diapause initiation, because only a few genes related to developmental arrest have been identified. The large percentage of unknown (novel) genes in the F library shows that diapause is a complex physiological process involving a number of unknown genes in the regulation of developmental arrest.

We also constructed an R library to identify specific genes expressed in nondiapause individuals. The up-regulated gene expression in nondiapause pupae identified from the R library usually corresponded to down-regulated expression in diapause-type pupae (Figure 3A), so these genes from the R library will help us to identify the genes associated with insect diapause if these differentially expressed genes in diapause-destined pupae are further characterized. A total of 150 sequences from the two libraries that were homologous to known genes were obtained. According to gene ontology analysis, most genes belonged to cellular process and metabolic process in the category of "biological process"; this implies that the insect brain at diapause initiation focuses on alteration of cellular and metabolic state. Signaling and transcriptional regulator activity also showed significant differences between the two libraries. Up-regulation of signaling genes and down-regulation of transcriptional regulators at diapause initiation indicate that signaling pathways are changed, global transcription levels are down-regulated, and diapause does require a unique gene expression regulatory mechanism.

The quality and reliability of the two SSH libraries were validated by investigating gene expression difference between diapause- and nondiapause-destined pupae. The two libraries were quite reliable, so the SSH method was useful to search for genes related to pupal diapause initiation. Subsequently, the expression patterns of four genes were detected by RT-PCR and Western blot analysis. All four genes were expressed higher at both the mRNA and protein levels during early pupal development in diapause-destined individuals than their nondiapause-destined counterparts. Apparently, these genes from the SSH library may reflect differential expression between diapause- and nondiapause-destined pupae for promoting diapause initiation.

Based on the functions of the putatively up- and down-regulated genes (Table 1), we have proposed a possible mechanism for diapause initiation.

## Changes in metabolism and energy

**Figure 5:**
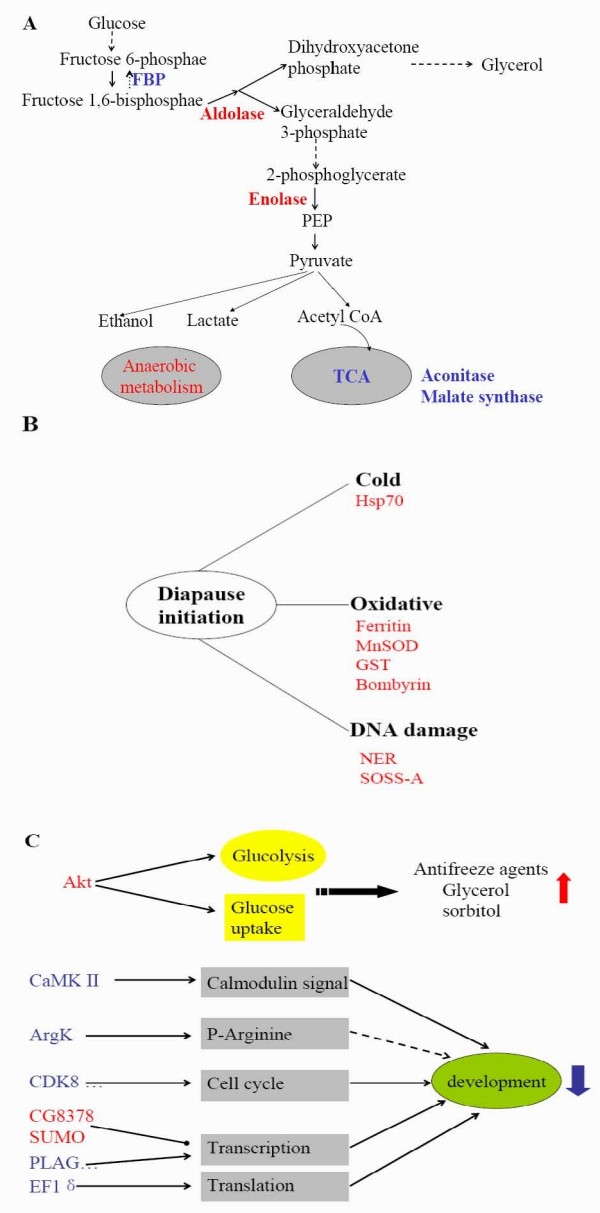
**Schematic representation of gene functions at diapause initiation**. Red represents up-regulated gene and blue represents down-regulated gene in diapause- destined individuals. (A) Changes in metabolism and energy at diapause initiation. (B) Gene expression changes in response to environmental stress. (C) Changes in signaling pathways at diapause initiation.

The brain of early diapause-destined pupae releases instructions to switch from development to diapause, so changes in metabolism and energy must be involved in the process. At diapause initiation, insects need to store energy and synthesize some specific compounds for cold hardiness, such as cryoprotectants. From the two SSH libraries, some transcripts function in metabolism and energy. Aldolase catalyzes the fourth step in glycolysis, which cleaves fructose 1, 6-bisphosphate and generates dihydroxyacetone phosphate and glyceraldehyde 3-phosphate. Dihydroxyacetone phosphate is synthesized into glycerol, and glyceraldehyde 3-phosphate enters the glycolytic pathway to generate energy. Enolase is an enzyme that catalyzes the ninth step of the glycolytic pathway, resulting in the formation of phosphoenolpyruvate (PEP) and pyruvate. FBP catalyzes the conversion of fructose-1,6-bisphosphate to fructose-6-phosphate, a key step between glycolysis and gluconeogenesis. To the best of our knowledge, gluconeogenesis and glycolysis are coordinated so that one way is relatively inactive while the other is highly active. As shown in Figure 5A, the down-regulation of FBP and up-regulation of aldolase and enolase suggest that gluconeogenesis diminished at diapause initiation, and glycerol biosynthesis is accelerated by glycolysis. Glycerol protects insects from cold stress. Meanwhile, the possible up-regulation of aldolase and enolase are responsible for generating pyruvate, which is also elevated in *S. crassipalpis *during pupal diapause [9,26], and pyruvate enters the glycolytic pathway to generate energy. Aconitase and malate synthase, which participate in the TCA cycle, are down-regulated. This result implies that the down-regulated aconitase and malate synthase may directly repress the TCA cycle. In diapause pupae of the flesh fly, *S. crassipalpis*, the TCA cycle is suppressed, and the metabolic intermediates from the TCA cycle are also reduced [9]. Therefore, inhibition of the TCA cycle and enhancement of glycolysis indicate that anaerobic metabolism is predominant at diapause initiation. In fact, respiration in diapause individuals is significantly lower than in nondiapause individuals [25], which is consistent with the decreased metabolic rate in diapause-destined individuals, and inhibition of the TCA cycle in the brain helps diapause individuals save energy. Enhancement of anaerobic metabolism has also been reported in recent studies of larval diapauses in the pitcher plant mosquito, *Wyeomyia smitbii *[27], and embryonic diapauses in the cricket, *Allonemobius socius *[28].

Additionally, three transcripts (HarDP-B843, HarDP-B170 and HarDP-B1356) for ATP generation were up-regulated at diapause initiation. ATP synthase f0 subunit 6 (HarDP-B843) plays a role in the production of ATP from ADP. Cytochrome *c *oxidase is a component of the respiratory chain in mitochondria. Cytochrome *c *oxidase subunit 2 (HarDP-B170) transfers electrons from cytochrome *c *to the bimetallic center of the catalytic subunit 1. Cytochrome *c *oxidase subunit 7C (COX7C, HarDP-B1356) is one of the nuclear-coded polypeptide chains of cytochrome *c *oxidase, the terminal oxidase in mitochondrial electron transport. Such a change of cytochrome *c *oxidase subunits during diapause has been reported in *C. pipiens *[29]. These observations suggest that energy demand still high during pupal diapause initiation.

As shown in Table 1A, the transcripts associated with lipid metabolism (apolipoprotein D and lipase) are down-regulated in diapause-destined pupal brain. Down-regulation of lipase has also been reported in early stage of diapause *C. pipiens*, but it is up-regulated in late diapause, suggesting that fatty acid-oxidation is suppressed in early diapause [30]. The down-regulation of apolipoprotein D and lipase implies that diapause individuals first utilize sugar as energy and store lipid for use during long diapause periods.

## Stress resistance

During the long overwintering phase, diapause pupae must encounter various stress challenges. The expressions of some specific genes are evoked in response to environmental stress [22], and stress resistance is important for the survival of diapause individuals. Hsp70 functions as a molecular chaperone to protect cellular proteins from denaturation in many species, including Diptera, Lepidoptera, Coleoptera and Hymenoptera [13]. In addition, a small hsp, Hsp21.4 identified by proteomic analysis, is more abundant in the brain of *H. armigera *pupae at diapause initiation [11]. Thus, the up-regulated Hsp70 at diapause initiation plays a role in cold-hardiness for overwintering (Figure 5B). Moreover, up-regulation of Hsp has also been reported under short day-length conditions, and Hsp up-regulation could represent a molecular exaptation to diapause [31].

Ferritin is the primary iron storage protein, and it functions in scavenging oxygen radicals [32,33]. Ferritin and ferritin light chain are up-regulated at diapause initiation, as reported in *Nasonia *[34] and in *S. crassipalpis *[8]. MnSOD is also up-regulated at diapause initiation. In *Caenorhabditis elegans*, MnSOD participates in the regulation of both longevity and dauer formation (a type of developmental arrest) as a physiological redox signaling modulators [35]. GST and bombyrin also have antioxidant function and are up-regulated in the brain, as reported in proteomic analysis of *H. armigera *[11]. Oxidative stress can damage tissues and cellular components during diapause. Therefore, the up-regulation of transcripts of antioxidant proteins will protect diapause individuals from oxidative stress (Figure 5B).

Rad23 is a nucleotide excision repair (NER) gene that, functions in DNA repair and protein degradation [36]. Integrator complex subunit 3, which is also called SOSS-A, is involved in sensing ssDNA and maintaining genome stability [14]. up-regulation of genes related to DNA repair (NER and SOSS-A) in diapause has not been reported previously. However, it is possible that DNA lesions occur under extreme environmental conditions during diapause, and the integrity of DNA is crucial for re-starting the development into an adult when diapause is terminated. Therefore, these up-regulated genes at diapause initiation mainly respond to stress resistance for insect survival in rigorous environmental conditions (Figure 5B).

## Signaling pathways

Genes involved in signaling pathway were also found in the SSH library (Table 1C). Akt is an essential component of the insulin signaling pathway for glucose uptake to synthesize sugar and also as an activator of the target of rapamycin (TOR) pathway to increase protein synthesis [37]. Insect organs and tissues need to accumulate a large store of sugar as energy and other substances, such as antifreeze agents glycerol and sorbitol, for use during a long diapause phase. Therefore, enhanced Akt transcription reflects increased sugar metabolism in diapause-destined pupal brain, and Akt participates in the regulation of energy reserves and in response to environmental stress at the onset of diapause (Figure 5C).

Calmodulin signaling, which is involved in the regulation of neuronal development and plasticity [38], is down-regulated at diapause initiation in *H. armigera *[11]. In this study, CaMK II, which modulates synaptic plasticity, learning, and memory [38], was down-regulated. ArgK was also down-regulated at diapause initiation, and high expression of ArgK, which is a developmental signal, was closely correlated with pupal development (data not shown). Thus, down-regulation of CaMK II and ArgK may cause developmental arrest at diapause initiation (Figure 5C).

## Cell cycle

During diapause, the cell cycle is arrested in the embryo of *B. mori *[39] and in the brains of *S. crassipalpis *[40] and *Chymomyza costata *[41]. Cyclin-dependent kinase 8 is a kinase partner of cyclin C, interacts with the large subunit of RNA polymerase II, and then participates in the regulation of the G1/S transition of mitosis [42]. More than 97% of the brain cells become arrested in the G0/G1 phase in the diapause pupae of *S. crassipalpis *[40]. Proteomic analysis of *Sitodiplosis mosellana *has found a strong up-regulation of inhibitor of nuclear factor kappa-B kinase interacting protein isoform 2 (IKIP2) during diapause, which contributes to inhibiting cell division during diapause [43]. Therefore, cyclin-dependent kinase 8 and five other transcripts down-regulated in the brain at diapause initiation may cause cell cycle arrest, inducing the insect to enter diapause (Figure 5C).

## Transcription and translation

Transcription and translation are two major energetic costs in cellular development. To reduce energy consumption, many genes are silenced during diapause [22]. In this study, several genes involved in the regulation of transcription and translation were identified (Table 1E). The down-regulation of transcription factor PLAG1 may result in the modulation of downstream target genes [44]. The down-regulation of elongation factor 1 delta indicates that translation is also suppressed at diapause initiation. In addition, some transcripts of proteins involved in transcription were up-regulated at diapause initiation: HarDP-C1098 is homologous to *Drosophila *CG8378, which contains the conserved MYND and SET domains found in human Smyd homologues. *Drosophila *Smyd represses transcription [45]. Smt3 (SUMO) is a reversible post-translational protein modifier that usually represses the activity of transcriptional activators [46]. Thus, we conclude that the down-regulation of PLAG1 and elongation factor 1 delta and the up-regulation of transcriptional repressors and SUMO lead to the global down-regulation of transcription and translation at diapause initiation (Figure 5C).

## Conclusion

In this study, differentially expressed genes at the early pupal stage of diapause- and nondiapause-destined individuals were isolated and identified that may be involved in regulation of diapause initiation. Diapause initiation is an intriguing developmental process with a complex molecular mechanism. Based on the above results, we suggest a possible molecular mechanism for diapause initiation. (1) Transition of metabolism and energy utilization. In addition to a decrease of metabolic activity, metabolic pathways are also changed in diapause-destined pupae at diapause initiation. Anaerobic metabolism predominates, and sugars and polylols accumulate in the brain. (2) Enhancement of stress resistance. The antifreeze agents glycerol and sorbitol as well as Hsp, GST, and others are heavily synthesized to protect the insect from rigorous environmental conditions. (3) Regulation of cellular development. The cell cycle is arrested, resulting in repression of pupal development toward adulthood. (4) Repression of transcription and translation. The up-regulation of transcriptional repressors, down-regulation of translational activators, and increased protein SUMOylation result in decreases of both gene transcription and protein translation at diapause initiation. This idea awaits detailed experimental investigation in the future.

## Materials and methods

### Animals

*H. armigera *larvae were reared on an artificial diet at 20°C with a L14:D10 (nondiapause type) and a L10:D14 (diapause type) photoperiod. After pupation, the two types of pupae were moved to the same conditions (20°C, L12:D12). Under these conditions, all nondiapause pupae developed toward adults, and more than 95% of diapause type pupae entered diapause. The developmental stages were synchronized at each molt by collecting new larvae or pupae. All tissues were dissected in insect saline containing 0.75% NaCl, and stored at -80°C until use.

### Suppression subtractive hybridization

We constructed two subtracted cDNA libraries (F and R) to detect high gene expression in diapause- and nondiapause-destined individuals at the early pupal stage (diapause initiation) using the PCR-Select™ cDNA Subtraction Kit (Clontech). In the F library, diapause type pupae were used as the "tester", and nondiapause pupa as the "driver". In the R library, the "tester" and "driver" were reversed.

After pupation, diapause- and nondiapause-destined pupae were incubated with the same condition 20°C and a short daylength (L12:D12) for 2-3 days before dissection. Total RNA from day 1-2 brains of diapause- and nondiapause-destined pupae was isolated using a guanidinium thiocyanate-chloroform method [47]. The mRNA was obtained according to the manufacturer's protocols of QuickPrep Micro mRNA Purification Kit (GE health).

Double-stranded cDNAs were synthesized from 1.0 μg of polyA+ mRNA and digested with RsaI to obtain shorter blunt-ended cDNA. The "tester" cDNA was subdivided into two populations, which were ligated to adaptor 1 and adaptor 2R, respectively. Two hybridizations were performed with the tester and driver cDNAs. In the first hybridization, the amount of driver cDNA was 25 times more than the tester cDNA. As a result, cDNAs that were not up-regulated were hybridized by driver cDNAs, only the up-regulated tester cDNAs were left as single strand. In the second hybridization, the amount of driver cDNA was 33 times more than the tester cDNA. As a result, the hybridized cDNAs were eliminated, leaving only the unhybridized cDNAs. The entire population of unhybridized molecules was then subjected to PCR to amplify target cDNA fragments (differentially expressed). Only the molecules of the tester sample, which were ligated to the two different adaptors, could be amplified exponentially. A second PCR amplification was performed using nested primers to get a low-background, high-level enrichment of the differentially expressed sequences.

The PCR products were analyzed by 2% agarose gel electrophoresis. Products from the secondary PCRs were inserted into pMD18-T by T/A cloning (TaKaRa). The recombinant plasmid DNAs were transformed into XL-1 blue competent cells. The DNA from recombinant clones was isolated and sequenced (Invitrogen).

### Bioinformatics analysis

All contigs and singlets were annotated according to the GO classification and the hierarchical structure using the Blast2GO suite [12]. The Blast2GO program, which assigns the GO terms based on the BLAST definitions, was applied with an E-value < 10^-5^. If a transcript was annotated with more than one GO category, it was split equally among them.

### RNA extraction and RT-PCR

Total RNA was extracted from the brain using the acid-guanidine method [47]. First-strand cDNA was synthesized using 1 μg of total RNA at 37°C for 1 h, with an M-MLV reverse transcription system (Promega, Madison, USA). The primers used to identify of differentially expressed transcripts by RT-PCR are presented in Additional File 4. The PCR reactions were subjected to 22--26 cycles consisting of 94°C for 30 s; 55°C for 30 s; 72°C for 1 min. Actin was used as an internal standard.

### Northern blot hybridization

Total RNA (25 μg) from the brain of day 1--2 diapause- and nondiapause-destined pupae was separated on a 1.2% agarose gel containing 0.22 mol/L formaldehyde, and transferred to a nylon membrane (Hybond N+, Amersham). Probes for hybridization were labeled with [α-^32^P]-dCTP using the Random Primer Labeling kit (Takara). After prehybridization for 4 h in 5× SSPE (1 × SSPE = 180 mM NaCl, 10 mM sodium phosphate, pH 7.7, 1 mM EDTA) containing 50% formamide, 5× Denhardt's solution, 0.1% SDS, and 100 μg/mL salmon sperm DNA, the radiolabeled probe was added and hybridization was conducted overnight at 42°C [48]. After hybridization, the membrane was washed in 0.2× SSPE at 42°C three times and exposed to X-ray film (Kodak) overnight at -70°C.

### Polyclonal antibody generation and western blot analysis

The ORFs of four genes (SUMO, MnSOD, sericotropin and TCTP) were amplified by PCR, using primers that contained restriction sites. The PCR product was digested by the appropriate restricted enzymes, then purified and subcloned into the pET28a vector. The recombinant pET plasmid was transfected into BL21 cells and induced by IPTG. The *E. coli *pellet was solubilized in 6 M urea in 50 mM Tris-HCl buffer, pH 8.0, followed by Ni-NTA column purification. Purified recombinant proteins were used to generate polyclonal antibodies in rabbit.

Proteins for western blotting were extracted from the brain and SG or the brain--SG complexes of pupae, quantified by the Bradford method [49], and stored at -80°C. Protein (20 μg) was separated by 15% SDS-PAGE and transferred onto an Immobilon-P Transfer membrane (Millipore). The immuno-reactivity was tested with antiserum, and then incubated with goat anti-rabbit IgG (Thermo), and protein was detected using the Novex Chemiluminescent Substrates (Invitrogen).

## Authors' contributions

WHX designed the research project, BB performed experiments. BB and WHX wrote the paper, and approved the final manuscript.

## Supplementary Material

Additional file 1 **Table S1 Checklist of known genes in the two libraries**. (A) Checklist of the sequences of known genes in the F library. (B) Checklist of the sequences those were homologous to known genes in the R library. All transcripts were compared to the sequences in GenBank using Blastx. Sequence description, minimum E-value and similarity are shown in the table. Click here for file

Additional file 2 Table S2 Comparison of the two libraries according to Gene Ontology criteria (Biological Process). Click here for file

Additional file 3 Figure S1 Classification of SSH ESTs according to Gene Ontology Criteria (Molecular Function and Cellular Component).
(A) Classification of SSH ESTs according to Gene Ontology criteria (Molecular Function). Gene Ontology analysis was carried out on the transcripts isolated from the two SSH libraries by Blast2GO program. The molecular Function combined graph was made based on ontology level 2. (B) Classification of SSH ESTs according to Gene Ontology criteria (Cellular Component). The cellular Component combined graph was made based on ontology level 2 by Blast2GO. Click here for file

Additional file 4 Table S3 Primer sequences used in PCR. Click here for file
