# Supplementary material for: Identification of gene expression changes associated with the initiation of diapause in the brain of the cotton bollworm, Helicoverpa armigera
Source: BMC Genomics. 2011 May 11;12:224. doi: 10.1186/1471-2164-12-224 (PMC3277317; doi:10.1186/1471-2164-12-224)
Supplement: Additional file 1 — Table S1 Checklist of known genes in the two libraries. (A) Checklist of the sequences of known genes in the F library. (B) Checklist of the sequences those were homologous to known genes in the R library. All transcripts were compared to the sequences in GenBank using Blastx. Sequence description, minimum E-value and similarity are shown in the table. [file 1471-2164-12-224-S1.DOC]

Table S1A

| **Diapause initiation up-regulated genes** | | | | |
| --- | --- | --- | --- | --- |
| **Seq. Name** | **Seq. Description** | **Length** | **eValue** | **Similarity** |
| HarDP-B81 | antifungal defensin ard1 | 376 | 2.79E-30 | 93.70% |
| HarDP-C253 | acylglycerol mitochondrial | 215 | 2.25E-16 | 74.65% |
| HarDP-B1016 | af393501_1antennal binding protein 8 | 514 | 1.39E-14 | 97.75% |
| HarDP-A55 | AGAP009642-PA | 136 | 7.03E-10 | 90.00% |
| HarDP-C349 | Aldolase | 1211 | 3.17E-149 | 87.15% |
| HarDP-B1564 | alpha-hemolysin | 1015 | 9.61E-04 | 69.00% |
| HarDP-A217 | antennal binding protein | 331 | 6.51E-35 | 72.00% |
| HarDP-A422 | arylphorin precursor | 163 | 1.33E-19 | 69.35% |
| HarDP-B843 | atp synthase f0 subunit 6 | 183 | 7.47E-15 | 94.70% |
| HarDP-C515 | autophagy-specific gene 4 | 473 | 2.25E-48 | 69.65% |
| HarDP-A112 | bombyrin | 330 | 1.98E-20 | 65.15% |
| HarDP-C649 | tnfrsf1a modulator | 607 | 8.95E-16 | 53.40% |
| HarDP-C309 | breast carcinoma amplified sequence 2 | 267 | 5.63E-07 | 64.20% |
| HarDP-C416 | c17orf39 homolog | 374 | 2.97E-48 | 90.80% |
| HarDP-C1098 | cg8378 cg8378-pa | 1056 | 4.59E-20 | 45.90% |
| HarDP-C1184 | chromosome 16 open reading frame 57 | 1142 | 5.66E-35 | 55.60% |
| HarDP-B170 | cytochrome c oxidase subunit ii | 656 | 1.94E-65 | 93.80% |
| HarDP-C290 | dienoyl | 248 | 8.26E-19 | 71.65% |
| HarDP-C496 | dihydrolipoamide dehydrogenase | 454 | 2.09E-62 | 77.20% |
| HarDP-C408 | dna replication licensing factor mcm9 | 366 | 2.57E-37 | 85.10% |
| HarDP-C323 | domain containing 3 | 281 | 3.25E-07 | 57.45% |
| HarDP-B1298 | elongation factor 1 beta | 543 | 5.73E-52 | 89.60% |
| HarDP-A209 | enolase | 325 | 9.73E-55 | 99.20% |
| HarDP-C556 | ferritin | 514 | 1.09E-51 | 82.00% |
| HarDP-C941 | ferritin light chain | 899 | 7.25E-74 | 72.15% |
| HarDP-A22 | glutathione s-transferase | 234 | 1.43E-10 | 57.05% |
| HarDP-B1451 | guanine nucleotide-binding protein gamma-1 subunit | 359 | 2.12E-14 | 92.50% |
| HarDP-A355 | heat shock protein 70 | 304 | 4.96E-51 | 98.70% |
| HarDP-C257 | hypothetical protein Phum_PHUM512530 | 215 | 3.25E-07 | 66.00% |
| HarDP-C1020 | misc_RNA | 978 | 4.32E-46 | 49.85% |
| HarDP-C509 | integrator complex subunit 3 | 467 | 3.28E-47 | 66.20% |
| HarDP-C197 | isoform a | 155 | 2.39E-05 | 59.75% |
| HarDP-B719 | kd orf | 1110 | 3.41E-29 | 63.65% |
| HarDP-C210 | methylthioadenosine phosphorylase | 168 | 9.84E-20 | 75.65% |
| HarDP-B1356 | mitochondrial cytochrome c oxidase subunit 7c | 647 | 1.19E-19 | 68.10% |
| HarDP-C604 | mitochondrial transcription termination factor | 562 | 3.09E-22 | 56.55% |
| HarDP-B1408 | mn superoxide dismutase | 624 | 2.60E-69 | 83.75% |
| HarDP-A596 | mrna turnover protein 4 mrt4 | 476 | 3.84E-32 | 75.65% |
| HarDP-C525 | myoneurin | 483 | 7.40E-15 | 46.70% |
| HarDP-C450 | nascent polypeptide associated complex protein alpha subunit | 408 | 1.84E-42 | 72.85% |
| HarDP-C441 | orf2-encoded protein | 399 | 1.49E-15 | 52.55% |
| HarDP-C195 | phosphatidate cytidylyltransferase | 153 | 1.54E-20 | 99.70% |
| HarDP-C924 | pol-like protein | 882 | 9.65E-15 | 46.50% |
| HarDP-C288 | popeye domain-containing | 246 | 2.92E-32 | 67.30% |
| HarDP-C230 | possible enoyl- hydratase echa12 | 188 | 2.48E-10 | 72.45% |
| HarDP-A58 | prophenoloxidase subunit 2 | 280 | 1.26E-22 | 89.65% |
| HarDP-C672 | rac serine threonine kinase | 630 | 1.70E-07 | 53.00% |
| HarDP-A345 | rad23 homolog b ( cerevisiae) | 400 | 9.49E-34 | 69.80% |
| HarDP-A46 | ras-related protein rab- | 232 | 1.87E-18 | 94.80% |
| HarDP-B15 | reverse transcriptase | 1026 | 1.08E-18 | 78.29% |
| HarDP-C1025 | ribosomal processing | 983 | 2.31E-148 | 79.40% |
| HarDP-A99 | ribosomal protein L12 | 187 | 2.24E-19 | 92.80% |
| HarDP-A62 | ribosomal protein L13 | 242 | 1.32E-24 | 92.95% |
| HarDP-C534 | ribosomal protein L14 | 492 | 2.02E-73 | 78.10% |
| HarDP-B1327 | ribosomal protein L20 | 247 | 2.74E-30 | 76.00% |
| HarDP-A37 | ribosomal protein L3 | 506 | 1.84E-69 | 95.30% |
| HarDP-B1282 | ribosomal protein L31 | 379 | 1.01E-48 | 94.95% |
| HarDP-B708 | ribosomal protein L34 | 285 | 2.02E-20 | 92.40% |
| HarDP-C306 | ribosomal protein L35 | 264 | 5.43E-26 | 90.25% |
| HarDP-C247 | ribosomal protein L6 | 205 | 7.29E-23 | 88.85% |
| HarDP-A81 | ribosomal protein L8 | 197 | 6.65E-24 | 91.65% |
| HarDP-A23 | ribosomal protein L9 | 138 | 1.28E-11 | 91.75% |
| HarDP-B87 | ribosomal protein rps21 | 195 | 1.95E-07 | 81.00% |
| HarDP-C445 | ribosomal protein s14 | 403 | 9.22E-34 | 99.30% |
| HarDP-B210 | ribosomal protein s30 | 390 | 9.05E-42 | 81.10% |
| HarDP-B1311 | ribosomal protein s5 | 236 | 3.29E-15 | 93.00% |
| HarDP-B1016 | sericotropin | 514 | 1.00E-14 | 97.75% |
| HarDP-C461 | splicing factor u2af large subunit | 419 | 2.00E-52 | 90.20% |
| HarDP-C808 | tbc1 domain family member 15 | 766 | 1.88E-42 | 52.40% |
| HarDP-A694 | translationally controlled tumor protein (TCTP) | 545 | 3.22E-79 | 93.45% |
| HarDP-A438 | type a von willebrand factor domain-containing protein | 235 | 6.15E-17 | 79.90% |
| HarDP-A589 | ubiquitin-like protein smt3(SUMO) | 517 | 5.91E-45 | 97.20% |
| HarDP-B1551 | unknown [Picea sitchensis] | 434 | 2.12E-14 | 91.67% |
| HarDP-B420 | F-box-like/WD repeat-containing protein TBL1X | 349 | 1.87E-26 | 72.50% |

Table S1B

| **Diapause initiation down-regulated genes** | | | | |
| --- | --- | --- | --- | --- |
| **Seq. Name** | **Seq. Description** | **Length** | **eValue** | **Similarity** |
| HarNP-776 | 26s protease regulatory subunit 7 | 734 | 1.95E-62 | 90.90% |
| HarNP-590 | 39s ribosomal protein mitochondrial | 549 | 5.20E-56 | 85.65% |
| HarNP-387 | 80 kda mcm3-associated protein | 345 | 5.45E-34 | 72.15% |
| HarNP-420 | accessory gland protein | 378 | 5.54E-63 | 93.25% |
| HarNP-475 | aconitase cg9244-pb | 475 | 1.62E-14 | 87.45% |
| HarNP-375 | alkylated dna repair protein alkb homolog 8 | 333 | 3.52E-22 | 78.85% |
| HarNP-740 | ankyrin repeat and mynd domain-containing protein 2 | 698 | 3.11E-59 | 65.65% |
| HarNP-1261 | apolipoprotein d | 1219 | 1.84E-88 | 61.35% |
| HarNP-423 | arg methyltransferase | 381 | 5.91E-57 | 90.15% |
| HarNP-138 | arginine kinase | 138 | 2.41E-10 | 99.70% |
| HarNP-872 | arginyltransferase 1 | 830 | 6.64E-07 | 71.26% |
| HarNP-433 | autophagy related protein atg4-like protein | 391 | 2.53E-68 | 75.95% |
| HarNP-449 | beta- -mannosyltransferase | 407 | 2.27E-24 | 58.85% |
| HarNP-1301 | ca2+ calmodulin-dependent protein kinase ii | 1260 | 1.66E-76 | 96.60% |
| HarNP-132 | cathepsin b | 132 | 8.31E-19 | 90.60% |
| HarNP-484 | cg7914 cg7914-pa | 442 | 2.31E-08 | 57.38% |
| HarNP-987 | coenzyme q6 homolog | 945 | 1.25E-95 | 65.75% |
| HarNP-342 | coiled-coil domain-containing protein 25 | 300 | 1.67E-43 | 87.75% |
| HarNP-297 | comparative gene identification 20 | 255 | 6.08E-17 | 64.25% |
| HarNP-328 | component of oligomeric golgi complex 8 | 286 | 2.93E-11 | 57.10% |
| HarNP-668 | cyclin-dependent kinase 8 | 626 | 1.20E-90 | 92.15% |
| HarNP-933 | double-stranded rna-binding zinc finger protein jaz | 891 | 2.22E-75 | 44.35% |
| HarNP-316 | drosophila melanogaster cg4645 | 274 | 6.15E-22 | 71.25% |
| HarNP-642 | elongation factor 1 delta | 642 | 3.49E-64 | 71.65% |
| HarNP-432 | elongation protein 3 homolog | 390 | 3.09E-66 | 96.70% |
| HarNP-765 | enoyl- isomerase | 723 | 2.01E-11 | 64.45% |
| HarNP-597 | fk506-binding protein | 555 | 1.33E-38 | 58.85% |
| HarNP-821 | fms interacting protein | 779 | 3.86E-51 | 60.90% |
| HarNP-1172 | fructose-1,6 -bisphosphatase | 1130 | ####### | 88.10% |
| HarNP-479 | golgi apparatus | 437 | 4.35E-44 | 69.40% |
| HarNP-284 | gtp-binding nuclear protein ran | 242 | 1.32E-40 | 99.00% |
| HarNP-448 | heat shock protein 70 -interacting protein | 406 | 1.47E-31 | 65.30% |
| HarNP-608 | hypothetical protein TcasGA2_TC012280 [Tribolium castaneum] | 566 | 5.05E-04 | 50.00% |
| HarNP-782 | isoform a | 740 | 1.39E-07 | 42.20% |
| HarNP-1012 | ku p80 dna helicase | 970 | 6.19E-21 | 46.55% |
| HarNP-554 | lacb2_drome ame: full=beta-lactamase-like protein 2 homolog | 512 | 1.88E-48 | 72.35% |
| HarNP-1246 | lipase | 1204 | 4.22E-61 | 57.80% |
| HarNP-412 | loc100170577 protein | 370 | 3.37E-34 | 82.35% |
| HarNP-363 | loc398534 protein | 321 | 3.43E-12 | 56.05% |
| HarNP-240 | low quality protein: tubulin alpha-3c d chain-like | 240 | 1.12E-39 | 100.00% |
| HarNP-491 | malate synthase | 449 | 1.57E-41 | 66.15% |
| HarNP-416 | mediator of rna polymerase ii transcription subunit 7 | 374 | 9.61E-31 | 77.80% |
| HarNP-591 | membrane lipoprotein | 549 | 5.46E-05 | 41.45% |
| HarNP-831 | minichromosome maintenance complex component 9 | 789 | 4.85E-65 | 65.75% |
| HarNP-323 | mitochondrial ribosomal protein L37 | 281 | 2.17E-19 | 67.40% |
| HarNP-503 | mitochondrial transcription factor | 461 | 6.04E-54 | 68.95% |
| HarNP-678 | mitotic checkpoint protein bub3 | 636 | 3.61E-29 | 82.25% |
| HarNP-713 | myelinprotein expression factor | 671 | 2.90E-27 | 72.65% |
| HarNP-441 | nadh-ubiquinone oxidoreductase fe-s protein 7 | 399 | 2.34E-61 | 93.25% |
| HarNP-1044 | neural precursor cell developmentally down-regulated 1 | 1002 | 3.43E-30 | 47.45% |
| HarNP-242 | n-myristoyltransferase 1 | 200 | 5.43E-26 | 87.65% |
| HarNP-282 | nonmuscle myosin essential light chain | 282 | 2.65E-20 | 91.60% |
| HarNP-389 | nucleotide binding protein 2 (nbp 2) | 347 | 3.33E-07 | 74.60% |
| HarNP-418 | oocyte zinc finger protein xlcof22 | 376 | 1.88E-10 | 55.05% |
| HarNP-1051 | phosphatidylethanolamine-binding protein | 1009 | 2.48E-15 | 52.90% |
| HarNP-233 | pih1 domain-containing protein 1 | 191 | 6.50E-11 | 65.60% |
| HarNP-730 | pleiomorphic adenoma gene 1 | 688 | 6.44E-13 | 48.00% |
| HarNP-331 | pleiotrophin-like protein | 331 | 5.76E-20 | 68.15% |
| HarNP-324 | potential nonsense-mediated decay helicase dbp2 fragment | 282 | 3.56E-33 | 78.90% |
| HarNP-779 | prefoldin subunit 4 | 737 | 3.72E-45 | 74.55% |
| HarNP-555 | protein saal1 | 513 | 3.20E-19 | 57.75% |
| HarNP-905 | Reptin | 863 | 1.44E-06 | 87.50% |
| HarNP-460 | ribosomal protein l11 | 418 | 4.90E-67 | 97.65% |
| HarNP-885 | serf-like protein | 843 | 7.51E-06 | 76.41% |
| HarNP-613 | sorting nexin | 571 | 7.58E-24 | 94.65% |
| HarNP-798 | transcription factor dp-2 (e2f dimerization partner 2) | 756 | 1.61E-46 | 74.05% |
| HarNP-515 | translin | 473 | 4.36E-68 | 72.35% |
| HarNP-893 | translocon-associated protein gamma | 851 | 1.61E-72 | 87.95% |
| HarNP-186 | tropomyosin 1 | 186 | 1.31E-11 | 97.45% |
| HarNP-393 | tubulin alpha-1 chain | 393 | 2.28E-64 | 99.00% |
| HarNP-473 | ubiquitin-conjugating enzyme e2 q | 431 | 1.04E-53 | 92.70% |
| HarNP-474 | uncharacterized conserved protein | 432 | 5.00E-40 | 64.75% |
| HarNP-472 | uroporphyrinogen decarboxylase | 430 | 3.58E-54 | 83.75% |
| HarNP-520 | williams-beuren syndrome critical region protein | 478 | 4.23E-31 | 62.15% |
