# Supplementary figures and images for: Identification of gene expression changes associated with the initiation of diapause in the brain of the cotton bollworm, Helicoverpa armigera
Source: BMC Genomics. 2011 May 11;12:224. doi: 10.1186/1471-2164-12-224 (PMC3277317; doi:10.1186/1471-2164-12-224)

**Supplementary Fig. 1**

**A Molecular Function**


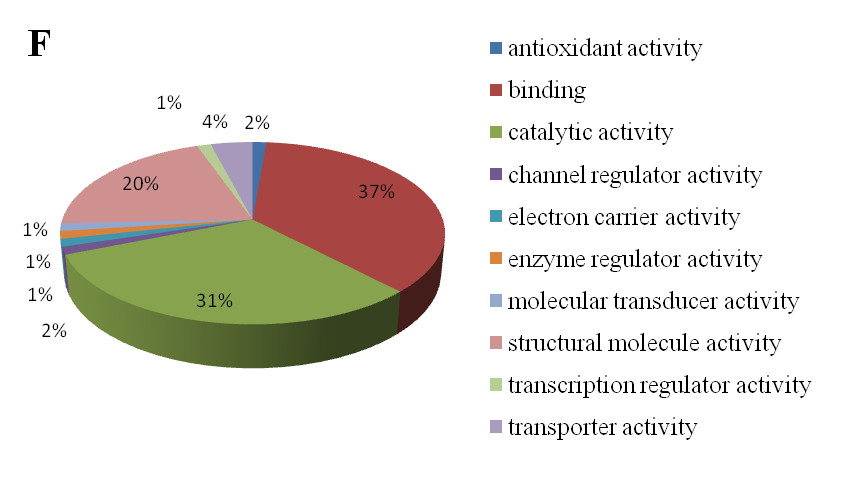


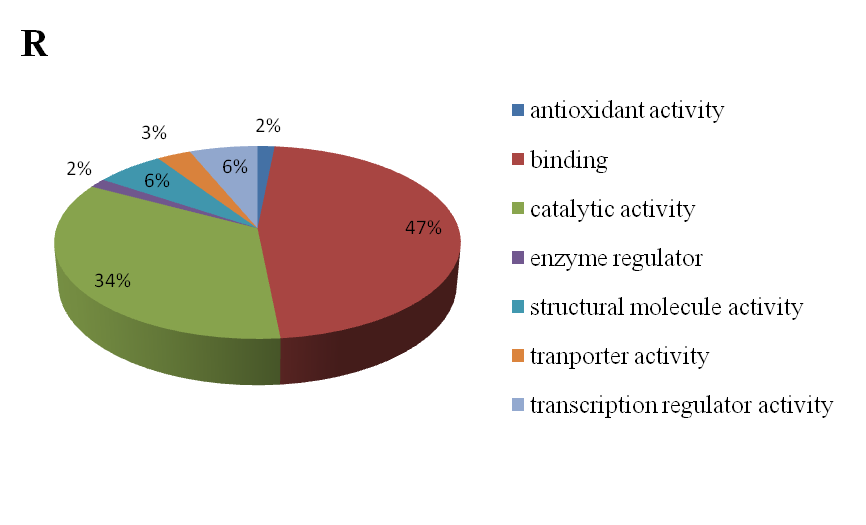


**B Cellular Component**


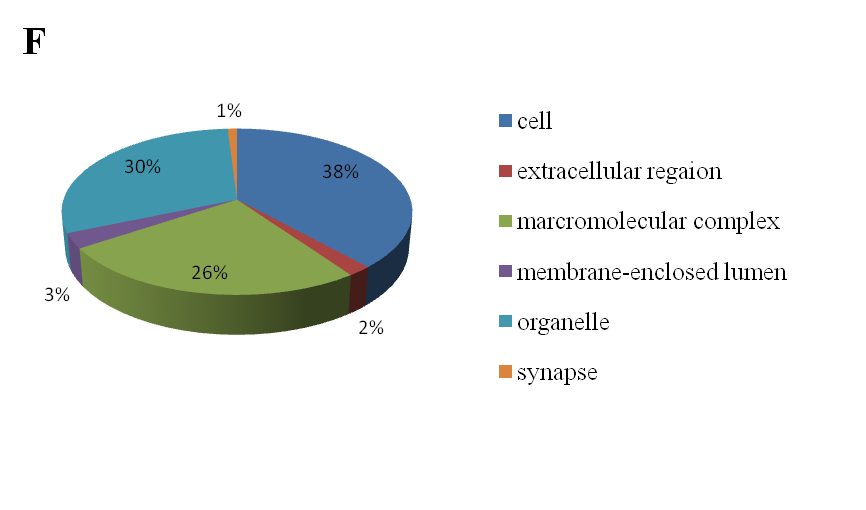


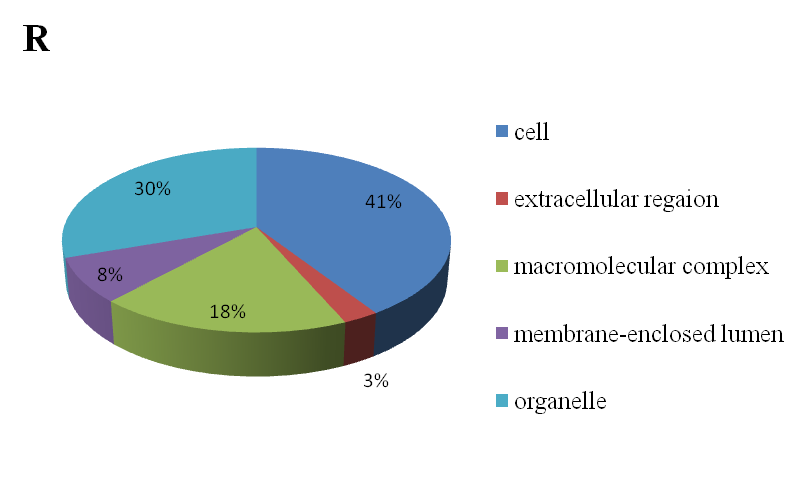

Supplement: Additional file 3 — Figure S1 Classification of SSH ESTs according to Gene Ontology Criteria (Molecular Function and Cellular Component). (A) Classification of SSH ESTs according to Gene Ontology criteria (Molecular Function). Gene Ontology analysis was carried out on the transcripts isolated from the two SSH libraries by Blast2GO program. The molecular Function combined graph was made based on ontology level 2. (B) Classification of SSH ESTs according to Gene Ontology criteria (Cellular Component). The cellular Component combined graph was made based on ontology level 2 by Blast2GO. [file 1471-2164-12-224-S3.DOC]
