# Supplementary material for: Identification of gene expression changes associated with the initiation of diapause in the brain of the cotton bollworm, Helicoverpa armigera
Source: BMC Genomics. 2011 May 11;12:224. doi: 10.1186/1471-2164-12-224 (PMC3277317; doi:10.1186/1471-2164-12-224)
Supplement: Additional file 4 — Table S3 Primer sequences used in PCR. [file 1471-2164-12-224-S4.DOC]

Supplementary Table. 2

| **Seq. Name** | **Primer** | **Primer sequence** | **Description** |
| --- | --- | --- | --- |
| HarDP-C534 | F | GTCGGTCATCTGTGCACGCTTC | ribosome protein L14 |
|  | R | CGTCTCACGTCAACAGCTCCG |  |
|  | | | |
| HarDP-C941 | F | GTCGTTCGCTACCAATCG | ferritin light chain |
|  | R | CGAGCTCATGCAGTTCCAC |  |
|  | | | |
| HarDP-C1020 | F | GGAAGTGCGTCTACTACT | misc_RNA |
|  | R | CCACTAGTAACAATCTAACGAG |  |
|  | | | |
| HarDP-C1192 | F | CAGGAGTCCAGAATCATCAC | NA |
|  | R | GCATACTCCTCCGAACCTG |  |
|  | | | |
| HarDP-A4 | F | CCCTGTATCGCGACGCTCCAC | NA |
|  | R | GTAGGGGTCGGCTGACGAGC |  |
|  | | | |
| HarDP-A694 | F | GGCACCTGACCAGGTAGATG | TCTP |
|  | R | CCATCGCAGTCCATGGACTC |  |
|  | | | |
| HarDP-B1016 | F | GGCCTCAGGTCTGACTGACG | Sericotropin |
|  | R | GACCAGCTGCTCATCCACCTTG |  |
|  | | | |
| HarDP-A112 | F | CACCCTGAATGGTGACGAGGTC | bombyrin |
|  | R | CGAGGAGCTTGGCAGCGTTG |  |
|  | | | |
| HarDP-B1408 | F | GCAGAACCTCTCGCCCAATGG | MnSOD |
|  | R | CTGCACACCCACAGATGCTGC |  |
|  | | | |
| HarDP-A589 | F | GCCAAGATAATGCTATCGTGCAG | SUMO |
|  | R | GCTGTCCGTCGAATCTGAACCG |  |
|  | | | |
| HarDP-C349 | F | CGCCCAGTACAAGAAGGAC | Aldolase |
|  | R | ACTGACCGCCAGACAGGA |  |
|  | | | |
| HarDP-C924 | F | CTTGGCATCAACACTAGGC | pol-like protein |
|  | R | CCCGTTGTTATAGTTTACGC |  |
|  | | | |
| HarNP-433 | F | GAGGAGAGAAAGCAGGCACC | Atg4 |
|  | R | TCAGACACCACGCAGTCCG |  |
|  | | | |
| HarNP-798 | F | GAGCAGTGAAACTGACTGGC | transcription factor dp-2 |
|  | R | GGCTATCTGCTGAACCATGC |  |
|  | | | |
| HarNP-138 | F | GTGAAAATGGGTGGCTG | arginine kinase |
|  | R | CTGATAACGCGAGATCG |  |
|  | | | |
| HarNP-893 | F | GGCACAATCAGGCTATCCG | translocon-associated protein gamma |
|  | R | CTCACTCACAATGCTGCCAC |  |
|  | | | |
| HarNP-779 | F | GATACTATGGCTAATTCGGG | prefodin |
|  | R | CACAGCTTCATCCCAGATG |  |
|  | | | |
| HarNP-1261 | F | GTTGTTGGCGATATGTGGG | apolipoprotein d |
|  | R | GCTCCTCGGTCCTTGAGA |  |
|  | | | |
| HarNP-1301 | F | TGTGCGACGTGCTGTGC | calmodulin-dependent protein kinase |
|  | R | GTCGGACACCAGATAGTG |  |
|  | | | |
| HarNP-905 | F | GGAGCTCACTCTCATATCC | Reptin |
|  | R | GGTGGTCTTCAGCGTCAG |  |
|  | | | |
| HarNP-1172 | F | CAATGACGCAGCAAGGAC | fructose-1,6 -bisphosphatase |
|  | R | TCGGAGACAAGCAGGCAC |  |
|  | | | |
| HarNP-1246 | F | CAAAGCCAGTGACTTGAGG | lipase |
|  | R | AACGACCGACACTGCGTC |  |
|  | | | |
| HarNP-423 | F | GGCACGGGCATCCTGTC | arg methyltransferase |
|  | R | GGCACGGGCATCCTGTC |  |
|  | | | |
| HarNP-503 | F | CTGCAGAAGAGAGGCTTGGC | mitochondrial transcription factor |
|  | R | CCTTAGATGAGATGCCAGGG |  |
|  | | | |
| Actin | F | CTGTACGCGTCCGGTCGTACC |  |
|  | R | GTAGTCTGTGAGGTCGCGGCC |  |
